# Acquired Surface Alexia in Spanish: A Case Report

**DOI:** 10.1155/2005/473407

**Published:** 2006-01-09

**Authors:** Aldo R. Ferreres, Macarena Martinez Cuitiño, Alicia Olmedo

**Affiliations:** ^1^Universidad de Buenos AiresArgentina; ^2^Hospital Eva PeronProvincia de Buenos AiresArgentina; ^3^CONICETArgentina

**Keywords:** Surface alexia, surface dyslexia, acquired dyslexia, dyslexia in Spanish, phonological reading, nonlexical reading

## Abstract

This paper reports a case study of acquired surface alexia in Spanish and discusses the most suitable tests to detect this syndrome in a writing system that is very regular for reading at the segmental and supra-segmental levels. Patient MM has surface alexia characterized by quantitatively good performance in reading words and pseudowords; accurate but slow and syllabic reading of words, nonwords and sentences; good performance in lexical decision tasks including words and nonwords; errors in lexical decision with pseudohomophones; and homophone confusions. This pattern of reading can be interpreted as a disorder in the lexical reading route and overdependence on the non-lexical route. We discuss nonlexical impairments and the interpretation of alexia and suggest tasks to identify surface alexia in a shallow orthography.

## 1. Introduction

Spanish is an ideal language to represent alphabetically. This is because of its primarily phonological properties which include as easy identification of phonemes, few vowels, strong syllabic segmentation, high incidence of CV and CVC syllables and few homophones allowing regularity in spelling to sound correspondences. Thus written Spanish can be considered a transparent alphabetic orthography compared to opaque alphabetic writing systems such as English and French.

The Spanish writing system contains 30 graphemes (25 single letters and 5 bigrams: <ch> <ll> <rr> <qu> <gu>) that correspond to 22 phonemes in the Rio de la Plata variety of Spanish. The writing system shows a tendency towards heterographic homophony; but not all varieties of Spanish have the same homophones, for example CASA and CAZA are homophones (pronounced /kasa/) in the Rio de la Plata variety of Spanish but not in Madrid. In the original Spanish written vocabulary, the correspondence between orthography and phonological word forms is almost perfect for reading but not for writing. Grapheme-phoneme correspondences are almost perfectly consistent so that mastery of approximately fifty rules (many univocal and other contextual) allows the reader to pronounce almost every phoneme correctly, even if the reader does not know the meaning or the pronunciation of a word [35, 37]. There are twenty-three graphemes – eighteen consonants and five vowels – that have a one-to-one correspondence with phonemes. Three graphemes have a pronunciation that depends on context-sensitive rules. These are adjacent vowel <C, G>, or position in the word <R>. Graphemes <GU> and <QU> are used only before vowels <E>, <I>; otherwise, <GU> is segmented as <G> + <U>. One grapheme represents two phonemes, <X>, and there is one grapheme that is not pronounced at all: <H>. <W>, used only in foreign words, is pronounced as either /b/ or /w/.

Spanish is completely regular for the reading aloud of vernacular words, which on the whole account for 99%

ISSN 0953-4180/05/$17.00 © 2005 – IOS Press and the authors. All rights reserved of the vocabulary. However, recent words that have not been adapted to Spanish as either calques or loanwords can be called exception words. In Spain, "*basketball*" has become "*baloncesto*" (calque) and "*baseball*" has been adapted into "*béisbol*" (loanword), after both words were introduced with their spelling in English. There are cases in which a single word becomes two in the target language, a calque and a loanword, with different meanings. For instance, "*tirebouchon*", from French, turned into "*tirabuzón*" (loanword) and "*sacacorchos*" (calque) in Spanish. In fact, many of these words are established in the orthographic lexicon, and some are frequent as they appear in advertising, sports, art, computers and technical/scientific jargon. No matter how regular these words are in their original language, most of them are irregular to the Spanish reader, and cannot be pronounced by applying orthography-to-phonology conversion rules.

The correspondences between phonemes and graphemes in Spanish are less consistent for writing. There are fourteen phonemes that are univocally associated to one grapheme: /a, e, o, u, ch, d, f, l, m, n, ñ, p, r, t/, eight phonemes that can be represented with two or three different graphemes, four whose graphemic representation is determined by conditional, context-sensitive rules /g, rr, i, k/, and four others with a further difficulty, since their graphemic representation depends on historical or etymological factors /b, s, x, y/, a case similar to the written presence of <H>, which does not stand for any phoneme but should be part of the spelling of many words.

The assignment of stress pattern is determined lexically in Spanish most of the time and phonological opposition between stressed and unstressed words exists /'xugo/[juice] vs. /xu'go/[he played]/. This is reflected in the orthographic distinction of the accent, a diacritic JUGO vs. JUGÓ [26]. In reading Spanish vernacular words, prosodic stress is governed by a highly restricted number of rules that are based on the specific characteristics of prosodic stress patterning. Polysyllabic words can be stressed on the last, the penultimate or the antepenultimate syllables (the resulting words being called oxytone, paroxytone and preparoxytone, respectively). Paroxytone words make up 74.6% of polysyllabic words in Spanish; 18.5% are oxytone; preparoxytone words represent 3.4%; and there is 0.1% of prepreparoxytone words. Stressed syllables are related to a word's final consonant. Most words ending in /n/, /s/ or a vowel are stressed in the penultimate syllable (paroxytone), and most words ending in all the remaining consonants are stressed in the ultimate syllable (oxytone). Only 17% of words require the diacritic accent, always indicating the stressed syllable, which is present only when a word seems to violate the natural stressing tendency. This is the case in all preparoxytone words, in oxytone words ending in /n/, /s/ or a vowel and in paroxytone words ending in any other consonant and in diphthongs. These tend to be part of the same syllable, and when they are not, they are stressed e.g. /'paria/paria vs. /pa'ria/paría. This explains why it is always possible to apply rules to determine which syllable should carry the prosodic stress when reading aloud in Spanish. If a word has an accent, the stressed syllable is signaled by orthography. However, if the word is not accented, then its stress pattern follows the natural tendency [24,35,37,39]. The accent can also distinguish orthographically between homophones from different grammatical categories. However, in that case, the distinction is not reflected in pronunciation e.g. EL article [the] vs. ÉL pronoun [he] or ESTE noun [este] vs. ÉSTE adjective [this].

The possibility that lexical stress patterns could be derived from rules does not imply that this is the way in which normal readers compute prosodic stress when they read aloud. If we assume that there is lexical reading even in transparent languages like Spanish, then we could argue that skillful normal readers know a word's spelling and retrieve its stress pattern in the same way. However, it can also be hypothesized that the stress pattern is retrieved later, while prosodic form is generated. This mechanism could be important for learning to read. For example, stored phonological representations are activated before orthographic representations are acquired, by decoding phonemes using orthography-to-phonology conversion rules [11].

How do these characteristics of the Spanish writing system impact on the functional architecture and typology of alexia? According to some authors [2–4, 13], transparency in Spanish orthography leads to a different typology and functional architecture "reading in Spanish is always mediated through phonology and language and it is even very difficult to figure out how to read without phonology" [3, p. 444]. "The existence of a double system of reading (phonological and semantic) would not then seem acceptable, as proposed for English . . . Our point, simply, is that reading in English and reading in Spanish represent quite different cognitive activities. Consequently, brain representation of written language and models for alexias and agraphias has to be somehow different." [4, p. 173]. Lastly, it is proposed that the characteristics of alexic disturbances will positively correlate with the idiosyncrasies of the respective writing systems [2].

However for others authors, transparency does not impact on the development of a lexical reading mechanism. “Every word in Spanish is also a unique string of letters that sounds a certain way and means something; therefore, Spanish speaking readers could, in principle, read known words lexically” [38, p. 92]. Several case studies of phonological [14,17,21,22] and deep alexia [15,20,33] in Spanish speaking patients have shown that alexic patterns with preserved lexical reading in this shallow language exist, in spite of severe disruption of non-lexical (phonological) reading mechanisms. This has been interpreted as evidence to support classical dual-route models of reading. The reverse dissociation, namely, impaired lexical and preserved non-lexical reading mechanisms, has received less attention in Spanish. Only one case of acquired surface alexia is published [25], although two others have been presented at meetings and reported in books [13,27,32, 38].

The difficulty in identifying surface alexia in shallow orthographies stems from the impossibility of finding a regularity effect on reading (poor irregular word reading), and the presence of regularization errors that are the two major symptoms in English and French speakers [12]. In Spanish, these symptoms cannot occur, since there are no irregular words. However surrogate tests of lexical reading can be administered. For example, reading tests with foreign words [25,32] and printed words presented without the graphic accent have been devised [25]. Heterographic homophony in Spanish is another way to identify surface alexia using homophone comprehension and visual lexical decision tests with pseudohomophones [10,11,30]. Also, little consideration has been given to the effects of syllabification and increased reading latencies in surface dyslexia. These phenomena can be considered alexic in Spanish if they appear in premorbidly fluent readers after brain damage.

It has been suggested that three types of surface alexia can be inferred from the classical dual-route model [18]. These three varieties are characterized by dissociations between reading of regular words and irregular words; “input” surface alexia would show this dissociation in word recognition and reading comprehension, “semantic” surface alexia would not present regular-irregular words dissociations in reading comprehension, whereas in the “output” variety, word recognition and comprehension would be intact. The absence of irregular words in Spanish poses a problem here too, but the tests mentioned above have the potential to identify these different types [40]. The non-lexical route for reading in Spanish has been uncovered with studies of phonological alexia [5,6,29]. However, in our opinion surface alexia in Spanish offers the possibility of revealing the operation of how the isolated non-lexical route in Spanish works when lexical processing in Spanish has been damaged.

In this paper, we aim to communicate a case of acquired alexia in Spanish whose reading pattern is the result of a dissociation between lexical and phonological reading mechanisms, a symptom compatible with input surface alexia as well as analyze the best tests to detect surface alexia in a shallow language such as Spanish and provide a more detailed account of the non-lexical route components in surface alexia.

## 2. Materials and method

### 2.1. Case details

MM, is a right-handed, male native speaker of Spanish who learned English and Portuguese as foreign languages. He has eighteen years of formal education and he worked as a lawyer and journalist. In August 2000, at the age of 53, MM suffered a closed cranial trauma with an initial coma. Recovery of consciousness revealed anterograde amnesia, which improved after a few days, and severe aphasia. Neurological assessment conducted four months after onset, revealed slight right-central facial paresis, right hemianopia and Wernicke’s aphasia with severe impairment of auditory and written comprehension. MM’s spontaneous speech was characterized by jargon, anomia, paraphasias and neologisms. Four months later, a CT scan showed a circumscribed lesion in the left temporo-parietal region. MM’s deficit improved and his response to rehabilitation was good. Eight months after the trauma, MM’s speech was no longer characterized by jargon and neologisms, his auditory comprehension had improved and his communication skills in everyday situations were acceptable. In spite of the recovery, the patient could not resume work because of his anomia, and his reading and writing difficulties. He reported that he “read very slowly” and that it was very difficult for him to understand written texts. Moreover, his writing, which used to be correct, was laden with mistakes stemming from the fact that he did not remember the spelling of many words. Language assessment with the Spanish version of *Battería per l’analisi dei deficit afasici* (BADA) [28] conducted eighteen months after trauma revealed the following: a) Auditory input tasks: preserved phonemic discrimination and auditory lexical decision with few errors in oral word-picture matching but impaired auditory grammaticality judgments, sentence comprehension and sentence repetition; b) Visual input tasks: few mistakes in non-word reading aloud, dictation and delayed copy (all errors were related non-words) with good performance in visual lexical decision, but performed slowly and always with subvocal pronunciation; few errors in word reading (formal and morphological errors), and therefore no effects of frequency, imageability, category or grammatical class; good performance in written word picture matching; moderately altered visual grammaticality judgments and slightly impaired sentence comprehension. All visual input tasks were performed after reading stimuli aloud or subvocally; c) Picture naming: several errors, especially with written words, with more difficulty in verb than noun naming; many semantic errors and few formal and morphological errors; d) Spontaneous oral production fluent but impaired with more difficulty in verb than in noun retrieval and considerable syntactic alterations, with neither phonetic errors nor syllabic simplification; e) Repetition of words and nonwords, preserved with few phonemic substitutions or transpositions; f) Short-term phonological memory span reduced with repetition of only two words. In sum, the patient was fluent, presented semantic and syntactic deficits, impaired word retrieval and reduced phonological span, alexia and agraphia, but sublexical transcoding was preserved (Appendix I). The characteristics of the patient's reading led us to postulate the existence of surface alexia, for although he read stimuli segments correctly, he did so by resorting to syllabification and subvocal pronunciation. His reading speed was slow for his level of education and profession. Moreover, he resorted to phonologically mediated reading in word, nonword and sentence reading, as well as in tasks that do not require pronunciation including visual lexical decision, written word – picture matching, visual grammaticality judgment and visual sentence comprehension.

### 2.2. Method

Two studies were conducted: The first between February and September 2002 (18 to 25 months after trauma) and the second in April 2005 (56 months after trauma). The goal of the second study was to use new tasks, but in order to avoid biased interpretations of the new findings some of the tasks from the first study were retested and will be referred to as first study tasks. In the results section, we report tests from the second study, which were also administered to five control subjects of the same age as MM who had the same level of formal education and no brain damage or acquired reading-writing or language impairment. MM's reading deficits were studied with tests of lexical reading and non-lexical reading to study components of the latter route and accented reading. The tests were: a) Word and nonword reading, repetition, dictation and delayed copy; b) Word type effects in reading; c) Visual lexical decision; d) Homophone comprehension; e) Stress/accent tasks; f) Non-lexical reading assessment tasks; and g) Dictation tasks.

*Task A*) *Word and nonword reading, repetition, dictation and delayed copy*

Word and nonword reading, repetition, dictation and delayed copy were assessed with Transpruebas Test, which contains 45 words and 45 nonwords [8]. Words are concrete imageable nouns controlled for length (15 of two, 15 of three and 15 of four syllables) and frequency (18 frequent and 27 infrequent words, with 6 and 9, respectively, for each length group). Syllabic complexity was also controlled (30 words containing a non-CV syllable and 30 containing CV syllables, with 5 and 10, respectively, for each length group). Thus, each length group (words of 2, 3 and 4 syllables) contained the following stimuli: 5 frequent words with CV syllables, 5 infrequent words with CV syllables, 1 frequent word with a non-CV syllable, and 4 infrequent words with a non-CV syllable. The list of non-words was built up by means of word syllable recombination, seeking non-words distant from actual words. They were matched with words in length and syllabic complexity. Word frequency values for this and all other tasks were obtained from Diccionario de Frecuencias de las Unidades Lingüísticas del Castellano [1]. Words whose occurrence exceeded 95/2,000,000 were classified as frequent and those whose occurrence fell below 15/2,000,000 were classified as infrequent. The same list of 45 words was used in reading, repetition, dictation, and delayed copying, denomination. The list of non-words was used for repetition, reading, dictation and delayed copying tasks. Because using the same stimuli in different tests is liable to introduce a distortion due to the learning effect, the list was divided into four parts and in each session a quarter of the list was taken for each task (for example the first quarter in repetition, the second in reading, the third in dictation, and the last in delayed copying); at the following session (separated by at least 4 days) the list was taken in another order (for example reading, dictation, delayed copying, repetition) and so on, until completing the 45 stimuli in each task and preventing any task in particular to be privileged in a special way by the use of the same stimuli as the previous task. Word and nonword reading was retested in the second study. In this study, reading speed was also measured from the moment the stimulus appeared on the computer screen to the moment the subject had finished pronouncing it. Five control subjects were also tested for this task.

*Task B*) *Words of different grammatical category including foreign words*

Word reading was evaluated with the BADA reading subtest, including different grammatical category word and foreign word reading tests. The BADA word reading subtest contains 92 tri-syllabic and tetra-syllabic stimuli, out of which 46 are medium-frequency words, and the other 46, low-frequency words belong to three different grammatical classes (20 nouns, 20 verbs and 20 function words), plus 16 concrete and 16 abstract nouns. A bisyllabic and trisyllabic word reading aloud test was prepared with 90 stimuli: 30 nouns, 30 verbs and 30 functional words. The frequency of nouns and verbs ranged between 30 and 40, whereas the functional words ranged from 40 and 1000 [1]. Reading time was measured. In order to make up for the absence of irregular words in Spanish, some researchers who have used foreign word reading tests [13,32]. We prepared a test containing thirty two French and English words that are frequently used in our linguistic region because they refer to trademarks or shop names, and whose pronunciation cannot be inferred by applying conversion rules e.g. boutique, Seven Up, Peugeot, Eveready.

*Task C*) *Visual lexical decision*

Visual lexical decision was evaluated with the BADA subtest containing 40 words (10 nouns, 10 verbs, 10 adjectives and 10 function words) and 40 nonwords obtained by changing a letter in the source word to an item with a different pronunciation. Reaction time was measured. To evaluate phonological mediation in visual lexical decision, we designed a test with 60 words, 30 nonwords and 30 pseudohomophonic nonwords. The nonwords were obtained by changing a letter in the source word into an item with a different pronunciation e.g. CAMISA (shirt) /kamisa/ → CAMIRA /kamira/. Pseudohomophonic nonwords were created by substituting a letter for another pronounced in the same way CAMISA /kamisa/ → CAMIZA /kamisa/. Thus, nonwords are not words in Spanish, whereas pseudohomophonic nonwords are not orthographic words in Spanish but they are phonological words. Words and source words had the same length and frequency, and all of them were nouns.

*Task D*) *Homophone comprehension*

A homophone comprehension test was devised to evaluate if meaning was accessed directly from orthography or via phonological mediation e.g. VASO (glass) /baso/ y BAZO (spleen) /baso/. The patient had to match a written word with its definition and reject the definition of its homophone. 28 pairs of homophones were selected, together with their definitions (56 definitions at all). One of the homophones in each pair was chosen and presented with both definitions; the test was carried out over two days, 28 stimuli each day. On the first day, the patient was given a definition followed by one of the homophones. For instance, the definition running “glass container used to drink” was followed by the word VASO “glass”. On the second day, the definition of the other homophone was presented, but it was followed by the same homophone as the previous day. In the example above, the definition was “bodily organ” and the word was VASO, instead of BAZO “spleen”. For each definition, the patient had to decide whether the word matched the definition. The answer was recorded as correct only when, for each pair, the patient chose the correct homophone and ruled out the incorrect homophone.

*Task E*) *Stress/accent tasks*

In order to assess orthographic knowledge of the diacritic, a visual accent decision task was devised, manipulating the presence of the accent. The task included 140 stimuli: 70 were words with their accent correctly placed and 70 were nonwords created by adding, deleting or changing the accent in the original word e.g. robot → róbot; mínimo → minimo; pálida → palída. The patient was asked to press one button if the accent was placed correctly and another button if it was placed incorrectly. The test was conducted under two different conditions: with an exposure time of 200 ms. and with no time limits. To evaluate MM’s capacity to sound out prosodic stress of orthographic stimuli, he was given a stress reading aloud task with 120 stimuli: 60 words spelled in lower case without an accent, e.g. oído → oido, razón → razon, unión → union; 30 nonwords with an accent and 30 nonwords without an accent e.g. tidió, bátur, cólosi; goriz, mante. More examples are provided in Appendix II.

*Task F*) *Non-lexical reading tasks*

In order to assess non-lexical reading, the patient had to perform to the following tasks: allograph matching, graphemic segmentation, orthography-to-phonology conversion rules, phoneme blending and a test of short term phonological memory. Allograph recognition was tested with a matching task in which the patient was presented with cards having three print letters, two of them being allographs (upper and lower case samples of the same letter) and the third one, a distractor (e.g. E – f – e). The patient had to point to the letter representing a different grapheme. The 27 single letters of the Spanish alphabet were tested. Graphemic segmentation was tested in a phoneme counting task. 18 cards with a three-lettered monosyllabic nonword were shown to the patient e.g. GUE /ge/ y PRI /pri/. In nine items, the letters represented two phonemes, whereas in the other nine, they spelled three phonemes. MM was asked to decide if the target nonword had two or three phonemes. Knowledge of orthography-to-phonology conversion rules was also tested in three different tasks, whereby the patient was asked to: i) sound out 28 graphemes, both single letters and bi-grams, corresponding to vowels and consonants; ii) sound out 23 graphemes, both single letters and bigrams, with the aid of a supporting vowel; only consonantal phonemes where used and the instruction was: "Read this letter as if it were followed by letter A"; and iii) read 36 syllables aloud, out of which 18 involved simple (univocal) conversion rules and 18 required application of complex (no univocal or context-sensitive) rules. As regards to phoneme blending, two tests were given in which the subject was asked to pronounce an item (a word or a nonword) from individually presented phonemes. The examiner dictated the constituent phonemes of each item one by one, such as /m-e-s/ and MM had to pronounce the whole item, /mes/ (month) in this case [5, 6,14,30]: i) syllabic blending of phonemes: 20 CV, 20 VC, 20 CVC; ii) blending of phonemes in words and nonwords 40 CVC sequences, 20 of them being words and the other 20, nonwords. Phonological short term memory was assessed with the digit span task from the Weschler Memory Scale.

*Task G*) *Writing to dictation*

To assess MM's agraphic writing, he was given a dictation task with 240 bisyllabic and trisyllabic words, 120 regularly spelled words e.g. mentón, moderna, puerto and 120 irregularly spelled words e.g. precio, hada, vencedor, half the stimuli were high-frequency words and the remaining low-frequency words (frequency was less than 10).

## 3. Results

*Task a*) MM read 98% of the words and 91% of the nonwords correctly, with no significant difference between stimuli types (see Table 1). There were no effects of frequency, length or syllabic complexity. He read the words slowly, first syllabifying and then pronouncing them as a whole, but stressed them correctly (see Table 1). The reading pattern with nonwords was similar but nonwords were not pronounced as a whole. Most mistakes were nonword approximations e.g. TRIJUDE /trixude/ → /tribude/; PANGARELLO /pangareyo/ → /bangareyo/ with the only mistake to words associated to stress errors PELÍCANO /pe'likano/ → /peli'kano/. In the word and nonword reading retest, MM produced only one error with words and one with nonwords. His reading speed was slower than controls, without a significant difference between words and nonwords. MM's performance was good in repetition and delayed copy tasks, and he performed somewhat worse in word and nonword dictation tasks, without significant differences between the two types of stimuli (see Table 1).

*Task b*) MM read 92% of the stimuli correctly, and there were no effects of frequency, grammatical class, concreteness or length on reading (see Table 1). Qualitatively, the reading pattern was similar to Task A. Out of seven errors, three were visual paralexias, two morphological paralexias and one was a stress error LEGAR /le'gar/ → /'legar/. On the reading of words belonging to different grammatical categories, MM showed an almost perfect performance, without category or grammatical class effects (see Table 1). In the foreign word reading task, MM read 22/32 (68.8%) of stimuli correctly. Errors were regularizations sometimes combined with phonological sequences COIFFEUR /kwafer/ → /kofer/. . . /koifer/. More examples are shown in Appendix II. The difference between oral reading of foreign words and Spanish words was significant (see Table 1).

*Task c*) MM showed excellent performance in visual lexical decision but response times were long. There were no differences in performance between words and nonwords (see Table 2). In visual lexical decision with pseudohomophonic nonwords, performance was better with words and nonwords (86.6% correct in both) than pseudohomophonic nonwords where performance fell to chance (46.6%). The difference between and pseudohomophone nonwords was significant (see Table 2). Most errors were false positives to pseudohomophonic nonwords. For example, pseudohomophones such as GUZANO correct form: GUSANO (worm). MM read

Table 1
Summary of Reading Tests

| *Task A Transpruebas test* | | | | | | | | | |
|---|---|---|---|---|---|---|---|---|---|
| | Words | | | | Nonwords | | | | Difference |
| | Accuracy | % | Response time Mean | SD | Accuracy | % | Response time Mean | SD | |
| *MM* | | | | | | | | | |
| Reading aloud | 44/45 | 97.8 | – | – | 41/45 | 91.1 | – | – | NS |
| Repetition | 45/45 | 100 | – | – | 39/45 | 86.7 | – | – | NS |
| Dictation | 40/45 | 88.9 | – | – | 36/45 | 80 | – | – | NS |
| Delayed Copy | 43/45 | 95.6 | – | – | 42/45 | 93.3 | – | – | NS |
| *MM (retest)** | | | | | | | | | |
| Reading aloud | 44/45** | 97.8 | 5173.7 | 2533 | 44/45 | 97.8 | 5507 | 1754.8 | NS |
| *Controls* | | | | | | | | | |
| Reading aloud | 225/225 | 100 | 717.9 | 189.8 | 225/225 | 100 | 984.4 | 278.4 | NS |
| Repetition | 225/225 | 100 | – | – | 225/225 | 100 | – | – | NS |
| Dictation | 224/225 | 99.6 | – | – | 225/225 | 100 | – | – | NS |
| Delayed Copy | 225/225 | 100 | – | – | 224/225 | 99.6 | – | – | NS |
| *Task B Words of different grammatical category* | | | | | | | | | |
| | | | Accuracy | | % | | | | |
| MM* | | | 88/90 | | 97.8 | | | | |
| Controls | | | 450/450 | | 100 | | | | |
| *Foreign words* | | | | | | | | | |
| MM* | | | 22/32** | | 68.8 | | | | |
| *Task E Stress reading aloud task**** | | | | | | | | | |
| MM* | | | | | | | | | |
| Words with deleted accent | | | 11/55 | | 20 | | | | |
| Nonwords with accent | | | 33/40 | | 82.5 | | | | |
| Nonwords without accent | | | 17/20 | | 85.0 | | | | |

*Retest (56 months after trauma).
**Foreign/Spanish words significant difference (Chi squared = 12.869; $p < 0.000$).
***Significant difference between words, nonwords with accent and nonwords without accent (Chi squared = 38.95; $p < 0.000$); non significant difference between nonwords with and without accent (Chi squared = 0.60; $p < 0.806$).
NS: non significant.

each stimulus syllabically, taking on average 15 seconds per item, regardless of whether they were words, nonwords or pseudohomophones. He was aware of the pseudohomophonic nature of some stimuli, which made him distrust his phonological strategy and thus increased his reading time and rate of errors with words in the list. MM's performance in the BADA visual lexical decision task could be explained by his using phonological reading mechanisms, since the task does not include pseudohomophones. In the second study, MM showed better performance with word and nonword stimuli than the first study, whereas performance with pseudohomophones worsened. Response times were considerably longer than for controls, and longest in the case of pseudohomophones (see Table 2). Controls did not show any significant difference between word, nonword and pseudohomophone stimuli.

*Task d*) MM performed at chance levels on homophone comprehension. He answered 12/28 (43%) of stimuli correctly, which is lower than expected, given his profession and formal education. He accepted both definitions for the same target homophone or discarded the correct definition while choosing the incorrect one.

*Task e*) MM performed at chance on visual accent decision with a limited exposure time (52.1% correct) with a similar proportion of false positive and false negative responses. In the stress test, MM looked at the stimulus for some time before pronunciation. His rate of correct answers for nonwords was high (with and without an accent) (see Table 2), suggesting that the capacity to compute prosodic stress by applying rules was intact. However in the case of words with the accent deleted, he pronounced 20% of the stimuli applying the right derivation rules. For the other 80%, he produced the pronunciation of the original word, a sort of "stress lexicalization" (see further examples in Appendix II).

*Task f*) MM performed well in allograph matching tasks and in phoneme counting with written nonwords, which suggests unimpaired letter recognition skills and an ability to segment letter string into graphemes. Performance was similar to controls (see Table 3). Out of three tasks designed to evaluate MM's mastery

Table 2
Summary of visual lexical decision tasks

| | | | | |
|---|---|---|---|---|
| *MM Visual lexical decision* (*BADA*) | | | | |
| | Accuracy | % correct | Reaction Time | SD |
| Words | 40/40 | 100 | 9681.5 | 3112.3 |
| Nonwords | 40/40 | 100 | 9829.3 | 5959.3 |
| Difference: NS | | | | |
| *MM visual lexical decision with pseudohomophones* | | | | |
| | Accuracy | % correct | | |
| Words | 52/60 | 86.6 | | |
| Nonwords | 26/30 | 86.6 | | |
| Pseudohomophones | 14/30 | 46.6 | | |
| Difference: S* | | | | |
| *MM visual lexical decision with pseudohomophones* (*retest*) | | | | |
| | Accuracy | % correct | Reaction Time | SD |
| Words | 56/60 | 93.3 | 7841.5 | 5739.7 |
| Nonwords | 27/30 | 90.0 | 7550.0 | 3450.5 |
| Pseudohomophones | 10/30 | 33.3 | 10178.5 | 6250.0 |
| Difference: S** | | | | |
| *Controls visual lexical decision with pseudohomophones* | | | | |
| | Accuracy | % correct | Reaction Time | SD |
| Words | 291/300 | 97.0 | 969.9 | 777.0 |
| Nonwords | 146/150 | 97.3 | 1055.5 | 602.5 |
| Pseudohomophones | 147/150 | 98.0 | 1055.3 | 751.0 |
| Difference: NS | | | | |
| *MM visual accent decision* | | | | |
| | Accuracy | % | | |
| Limited time (200 ms) | 73/140 | 52.1 | | |
| Without time limitation | 96/140 | 68.6 | | |

*Significant difference between W, NW and PSH Chi squared = 10.800 $p < 0.001$.
**Significant difference between W, NW and PSH Chi squared = 44.875 $p < 0.000$; significant difference between NW & PSH Chi squared = 20.376 $p < 0.000$; significant difference between W & PSH Chi squared = 36.818 $p < 0.000$; non significant difference between W & NW Chi squared = 0.310 $p = 0.578$.
Note: PSH, W, NW, S, NS: pseudohomophones, words, nonwords, significant, non significant.

of orthography-to-phonology conversion, his performance was most impaired when asked to sound out isolated graphemes (only 53.6% of correct answers). 12/13 errors were vowel additions e.g. <ñ> /ñ/ > /ñe/; <ch> /c/ > /ci/, a pattern which suggests he had access to the phoneme but could not pronounce it in isolation and thus added a vowel. He performed better when asked to sound out graphemes with supporting vowels and in the syllable sounding out tasks (91.3% and 80.6% of correct answers, respectively). Only 10 of the errors made in these three tests were specific failures in application of grapheme-phoneme conversion rules, and only three rules, two complex (<J> and <G> roules) and one simple (<Z>), were involved. These results suggest a slight alteration of conversion skills, affecting a few rules only, and disruption in phonological manipulation skills. MM performed significantly worse with nonwords (60%) than words (95%) in blending tasks. In all the tasks retested, MM's performance improved and the pattern preserved (see Table 3). In the nonword blending test with different syllabic structures, the patient performed poorly, but a pattern emerges in terms of syllabic structure (CV: 65%, VC: 80%, CVC: 5%). The low performance with CVC syllables (5%) contrasts with the high rendering of VC syllables (80%), as well as CVC syllables in the previous test (60%). The thirty errors consisted of twenty five phonemic mistakes (substitution, addition or omission phoneme), four lexicalizations (e.g. /n-a-r/ > /mar/ SEA; /t-o/ > /tu/ YOU) and one omission. The analysis of the phonemic mistakes reveals a primacy effect. MM tended to preserve initial phnemes and failed (substituted or omitted) with syllabic codas (Appendix II). The same pattern was observed in the retest of this task, although he showed a quantitative improvement. The results suggest that short-term memory and phonological manipulation skills are impaired. Despite the metalinguistic nature and the difficulty of both blending tasks (and also of the grapheme sounding out test), control subjects made few mistakes (see Table 3). MM's digit span was three and his indirect digit span was two.

*Task g*) MM wrote 109/120 (90.8%) of regular words and 81/120 (67.5%) of irregular words correctly, with a significant difference in performance (Chi squared = 19.80, $p < 0.001$). He hesitated with irregular stimuli so that on 46 items, he wrote two or three phonologically plausible variants of an irregular word before producing an answer e.g. /xubentud/ (JUVENTUD) → JUVENTUD, JUBENTUD (examples in Appendix II). Most of the irregular word errors (33/39) were phonologically plausible spellings, and 9/11 errors for regular words had a morphological origin e.g. omission of plural affix −s. MM's performance in writing to dictation was less than expected in terms of profession and formal education. It matches the pattern called surface agraphia in English [40].

## 4. Discussion

We will discuss MM's alexia in terms of lexical-nonlexical reading routes, including accent reading, the nature of MM's nonlexical reading impairments and implications of nonlexical impairments for the interpretation of other types of alexia, and the most suitable tasks to identify surface alexia in a shallow language like Spanish.

Words and nonwords were read with the same accuracy, speed and syllabification by MM and without lexical effects of frequency, word category or concreteness. The main difference in reading of words and nonwords was that words seemed to activate lexical phonological representations which allowed their pronunciation as a whole, and to guess their ending which in a few stimuli resulted in visual or morphological paralexias. The absence of a length effect can be explained by MM's strategy of syllabified reading. He made no regularization errors in reading tasks because Spanish has no irregular words. However he produced regularization errors with foreign words and his performance was significantly worse with foreign words than with Spanish words. Foreign word reading has been used as a proxy for irregular words in Spanish since they do not conform to the spelling to sound rules of the language. However, their results are difficult to interpret given that foreign word pronunciation and familiarity differs across socio-cultural contexts. MM's foreign word reading is qualitatively similar to surface alexia in opaque languages and we consider that this is a significant reading impairment for him when one takes into account his acquaintance with several foreign languages.

In our opinion these data indicate a severe impairment to lexical reading in Spanish together with a relatively preserved non-lexical reading route and a subsequent overdependence on the non-lexical reading for word and nonword reading. Due to the linguistic properties of written Spanish, the phonological mediation implied in non-lexical reading can be used to perform visual lexical decision as well as tasks requiring access to meaning, *insofar* as stimuli are not homophones or pseudohomophones. MM's performance in visual lexical decision (the BADA subtest) and written word-picture matching (object comprehension and action comprehension of the BADA subtests), was perfect, although slow. On the other hand, he performed at chance in visual lexical decision with pseudohomophones and in homophone comprehension. This pattern leads us to characterize his impairment as input surface alexia that is caused by a breakdown in access from visual input to the orthographic lexicon. Thus, visual lexical decision with pseudohomophones and homophone matching tasks are crucial to the identification of orthographic lexical impairment in Spanish, and particularly useful for the diagnosis of input surface alexia.

In dual-route models of reading, lexical prosodic stress can be retrieved through two different mechanisms, at least at the theoretical level: by activating the word's phonological representation through the lexical route (via input orthographic lexicon – semantic system – output phonological lexicon) or by activating the same phonological representation through the non-lexical route, (retroactive activation at the phonological buffer from phonemes obtained by sublexical conversion). MM's impaired performance in visual accent decision with limited time, visual lexical decision with pseudohomophones and homophone comprehension tests support our contention that the lexical route is not available, indicating a severe disruption to the orthographic input lexicon. The non-lexical route is available, and results in deleted accent word reading tasks showing the sequence of phonemes obtained through non-lexical reading is enough to activate the word's phonological representation (including stress) in spite of the diacritic's deletion in the stimulus, which is a sort of stress lexicalization.

On the other hand, in accented and unaccented nonword reading, MM did not place the stress at random. In 80% of the stimuli, he stressed syllables prosodically

Table 3
Non-lexical reading related tasks

| | MM | | MM (retest) | | Controls | |
|---|---|---|---|---|---|---|
| | Accuracy | % correct | Reaction Time | SD | | |
| Allograph recognition | 27/27 | 100 | 123/125 | 98.4 | | |
| Phoneme counting | 17/18 | 94.4 | 89/90 | 98.9 | | |
| *Sound out* | Accuracy | % correct | Accuracy | % correct | Accuracy | % correct |
| Graphemes | 15/28 | 53.6 | 30/44 | 68.2 | 218/220 | 99.1 |
| Graphemes with supporting vowel | 21/23 | 91.3 | 41/42 | 97.6 | 210/210 | 100 |
| Syllables | 29/36 | 80.6 | 42/44 | 95.5 | 220/220 | 100 |
| *Phoneme Blending* | | | | | | |
| Phoneme in syllables | 30/60 | 50* | 86/120 | 71.7 | 284/300 | 94.7 |
| Phoneme in CVC W and NW | 31/40 | 77.5** | 186/200 | 93.0 | | |
| *Phonological short term memory* | | | | | | |
| Direct digit span: 3 | | | | | | |
| Indirect digit span: 2 | | | | | | |

*(CV 65%, VC 80%, CVC 5%) Significant difference (Chi squared = 25.200; $p < 0.000$).
**(NP: 60%; P: 95%) Significant difference between words and nonwords (Chi squared = 7.025; $p < 0.008$).

according to the correct derivation rules. This suggests existence of a non-lexical mechanism of stress derivation that is implicit. In fact, if stressing *accented* nonwords can be considered the result of the conscious application of stress rules, this does not hold for the other two rules used by MM to pronounce *unaccented* nonwords: stressing nonwords ending in /n/, /s/ or a vowel in the penultimate syllable and stressing nonwords ending in all the other consonants in the last syllable. This could be the only available mechanism for Iribarren's patient [25], a surface alexic who, unlike MM, made no stress lexicalization errors in deleted accent word reading. The data here are not conclusive enough to corroborate this hypothesis, but patients with surface alexia and a severe impairment of the output phonological lexicon seem likely to show this pattern. The absence of stress lexicalization may also be a distinctive symptom of output surface alexia in Spanish.

Finally, the fact that MM's performance in visual accent decision task with no time limits is better than tasks with a limited time can be explained in terms of stimulus persistence giving MM time to retrieve phonological representations and check whether stimuli violate orthographic rules. On the whole, the results from the stress tests suggest that MM resorts to a mechanism which does not rely on the input orthographic lexicon but on the interaction between the non-lexical route and the output phonological lexicon. This mechanism may depend on a set of implicit rules other than orthography-to-phonology conversion rules, since they take into account the number of syllables, the presence and position of the accent, and the type of final phoneme.

In spite of good performance in nonword reading, the processing components of the non-lexical reading route were not completely intact. MM's results in non-lexical reading tasks suggest that letter recognition skills and the ability to segment letter strings into graphemes are unimpaired whereas grapheme-phoneme conversion skills are slightly impaired, affecting a few rules only and phonological short term memory and phonological manipulation skills are severely impaired. Given phonological short-term memory and phonological manipulation difficulties, how can MM read syllables and nonwords adequately? Blending tasks consist of ordering the string of phonemes and organizing them into a syllabic structure [7,38]. It is likely that the sequence of graphemes in a written stimulus provides more information to organize the syllabic structure than the sequence of phonemes when read one at a time. Another possibility is that MM segments nonwords as orthographic syllables, which he processes one by one by means of the orthographic-phonological conversion of *complete syllabic units* [34, 36,37]. That way he avoids memory buffer overload and bypasses blending difficulties. This strategy can be highly successful in a language like Spanish.

The dissociation between the phonological deficit and good non-word reading is relevant to another issue. A high proportion of *phonological* alexic patients have deficits in phonological tasks such as blending and phonological short term memory [6,9,29]. This frequent association between phonological and impaired non-lexical reading deficits can be taken as evidence that, according to some interpretations, phonological alexia is due to general phonological weakness affecting nonwords [19,23,29,31]. However MM's difficulties in phonological manipulation and phonological short memory are not enough to produce the disruption of *all* non-lexical reading mechanisms, let alone produce the phonological alexia pattern. In other words, poor performance in blending and phonological short memory is not sufficient to produce the difficulties in nonword reading that characterize phonological alexia, at least in MM.

In summary, we contend that surface alexia is underreported in Spanish because some of the characteristics of the reading system are overlooked and clinical tests to detect problems with lexical-semantic reading ability are not administered. Spanish has a very shallow orthography at the segmental and suprasegmental level for oral reading and this allows correct non-lexical reading of words and nonwords, good performance in visual lexical decision and preserved comprehension via phonological mediation. However, the evidence from MM shows that surface alexia in Spanish can be diagnosed on the basis of quantitative and qualitative patterns of word and non-word reading, false positive errors in lexical decision with pseudohomophones and homophone confusion. Accent visual decision and accent reading tasks can offer additional evidence to identify surface alexia subtypes in Spanish. Foreign word reading can show regularization errors that are not noticed in Spanish reading. Dictation of Spanish words like JUVENTUD, SERVICIO which are regular for reading but irregular for writing, show the presence of surface agraphia which is frequently reported in surface alexic patients. Moreover, good nonword reading distinguishes surface alexia from phonological and deep alexia, syndromes in which nonword reading is severely disrupted. Recent evidence shows that typical alexia patterns can be observed in shallow languages like Spanish [14–16,20–22,25,30,33,38]. The particular characteristics found in Spanish alexic patients can be accounted for by the assumption that the same functional architecture works with different language specific codes and does not support the postulation of a functional architecture with significant cross-linguistic differences.

## Acknowledgements

This paper was support by UBACYT P002 grant. We thank numerous people for valuable assistance, comments and criticisms: Sergio Dansilio, Silvia Jacubovich, Cynthia López, Silvia Mazabel, Maximiliano Wilson, Angela Signorini and Francisco Valle-Arroyo. We thank MM for his patience and kind collaboration.

## Appendix I.

**MM Language Evaluation using BADA [25].**

| **Nonlexical tasks** | Accuracy | % correct |
|---|---|---|
| Phoneme discrimination | 60/60 | 100 |
| Auditory-visual matching | 58/60 | 97 |
| Nonword repetition | 33/36 | 92 |
| Nonword reading | 43/45 | 96 |
| Nonword dictation | 22/25 | 88 |
| Nonword delayed copy | 13/15 | 87 |
| **Lexical-Semantic Tasks** | | |
| Auditory lexical decision | 80/80 | 100% |
| Visual lexical decision | 77/80 | 96% |
| Word repetition | 39/45 | 87% |
| Word reading | 86/92 | 93% |
| Word dictation | 40/46 | 87% |
| Word delayed copy | 15/16 | 93% |
| Object oral picture naming | 16/30 | 53% |
| Action oral picture naming | 0/4 | 0% (suspended) |
| Object written picture naming | 11/22 | 50% |
| Action written picture naming | 0/4 | 0% (suspended) |
| Object auditory comprehension | 37/40 | 92% |
| Action auditory comprehension | 20/20 | 100% |
| Object visual comprehension | 38/40 | 95% |
| Action visual comprehension | 19/20 | 95% |
| **Sentence Processing Tasks** | | |
| Auditory grammaticality judgement | 39/48 | 87% |
| Visual grammaticality judgement | 19/24 | 79% |
| Sentence repetition | 2/14 | 14% |
| Phrase repetition | 3/6 | 50% |
| Phrase reading | 6/6 | 100% |
| Auditory Sentence comprehension | 51/60 | 85% |
| Visual Sentence comprehension | 43/45 | 96% |
| **Memory Tasks** | | |
| Word Recognition | Correct Lists | |
| 4 stimulus | 22/24 | 92% |
| 6 stimulus | 21/24 | 87% |
| 8 stimulus | 18/24 | 75% |
| Nonword Recognition | | |
| 4 stimulus | 23/24 | 96% |
| 6 stimulus | 18/24 | 75% |
| Word Reproduction | Correct Lists | |
| 2 stimulus | 7/10 | 70% |
| 3 stimulus | 0/10 | 0% |
| 4 stimulus | 0/5 | 0% |
| Nonword Reproduction | Correct Lists | |
| 2 stimulus | 3/10 | 30% |
| 3 stimulus | 0/5 | 0% (suspended) |
| 4 stimulus | 0/5 | 0% (suspended) |

## Appendix II.

**1) Sample of errors in Reading tasks.**

**BADA test**

| | |
|---|---|
| Words | |
| ACEPTAMOS /aseptamos/ → /aseptado/ | MPL |
| ENVIDIABA /enbidiaba/ → /enmidiaba/ | RNW |
| VOLCARON /bolkaron/ → /mulkaron/ | RNW |
| BUFANDA /bufanda/ → /mufanda/ | RNW |
| DEFENDÍA /defendia/ → /demensia/ | VPL |
| CONFUNDEN /konfunden/ → /ponfunden/ | RNW |
| Nonwords | |
| GU /gu/ → /xu/ | RNW |
| GA /ga/ → /xa/ | RNW |

MPL, VPL, RNW: morphological paralexia, visual paralexia, related non-word.

**Transpuebas test**

Words

| | |
|---|---|
| PELÍCANO /pe'licano/ /peli'cano/ | stress error |

Nonwords

| | |
|---|---|
| PANGARELLO /pangareyo/ → /bangareyo/ | RNW |
| MUGUETA /mugueta/ → /mugueto/ | RNW |
| JIPELLO /xipeyo/ → /xipelo/ | RNW |
| TRÍJUDE /trixude/ → /tribude/ | RNW |

**Words of different grammatical category reading**

| | |
|---|---|
| AQUEL /akel/ → /akuel/ | RNW |
| BUQUE /'buke/ → /bu'ke/ | stress error |

**Foreign words**

| Stimulus | Expected pronunciations | MM answers |
|---|---|---|
| BOITE | /buat/ | → /boite/ |
| LIGTH | /lait/ | → /lig/ |
| TUPPERWARE | /taperwea/ | → /tuperguare/ |
| ROUND | /raund/ | → /run/ |
| FREEZER | /fri:se/ | → /fresar/ |
| SPRITE | /sprait/ | → /sprits/ |
| COIFFEUR | /kuafer/ | → /cofe/ |
| RENAULT | /reno/ | → /renoult/ |
| NYLON | /nailon/ | → /nilon/ |

**2) Sample of errors in visual lexical decision tasks.**

**Visual lexical decision**

Bada test*

| | |
|---|---|
| EXPRORAR | false positive |
| CAÑUELO | false positive |
| DABAJO | false positive |

Source words: EXPLORAR, PAÑUELO, DEBAJO

*First evaluation.

**Visual lexical decision with pseudohomophone nonwords***

**Pseudohomophone nonwords**

| | |
|---|---|
| PANTAYA | false positive |
| PRESIO | false positive |
| BALLE | false positive |
| VARRIO | false positive |
| PRECIÓN | false positive |
| PASAGES | false positive |
| BESTIDO | false positive |
| NARIS | false positive |
| GANANSIA | false positive |
| HAULLIDO | false positive |
| HARTISTA | false positive |
| VUFANDA | false positive |
| PINSEL | false positive |
| GUZANO | false positive |
| VOTELLA | false positive |
| SORRO | false positive |
| AJENDA | false positive |
| ZAPO | false positive |
| RELIJION | false positive |

Source words: PANTALLA, PRECIO, VALLE, BARRIO, PRESIÓN, PASAJES, VESTIDO, NARIZ, GANACIA, AULLIDO, ARTISTA, BUFANDA, PINCEL, GUZANO, BOTELLA, ZORRO, AGENDA, SAPO, RELIGIÓN.

**Common nonwords**

| | |
|---|---|
| ILIS | false positive |
| INTANCIA | false positive |

Source words: IRIS, INFANCIA.

**Words**

| | |
|---|---|
| PABELLÓN | false negative |
| ZURCO | false negative |
| BEATA | false negative |
| HIGUERA | false negative |

*Second assessment (56 months after cranial trauma).

**3) Sample of errors in Phoneme Blending in syllables***

CVC syllables
/f-i-k/ → /fit/
/f-o-l/ → /foi/
/l-i-m/ → /lia/
/m-o-n/ → /mob/
/x-u-s/ → /xui/
/n-e-r/ → /mer/
/m-o-n/ → /mou/
/x-o-r/ → /xur/
/k-o-r/ → /kar/
/f-i-k/ → /fip/
CV syllables
/k-u/ → /ki/
/n-u/ → /ni/
/n-e/ → /be/
/x-u/ → /gu/
/x-o/ → /xu/
VC syllables
/on/ → /om/
/ic/ → /ip/
/um/ → /un/

*Second assessment (56 months after cranial trauma).

**4) Sample of errors MM accent reading aloud, stimulus and answers examples.**

**Nonword with and without accent reading.**

| Stimulus | Correct pronunciation | MM answers | Score |
|---|---|---|---|
| CÓFIRA | /'kofira/ | /'kofira/ | C |
| MANTÉ | /man'te/ | /man'te/ | C |
| GÁDUCEL | /'gadusel/ | /'gadusel/ | C |
| TORUDO | /to'rudo/ | /to'rudo/ | C |
| BLATUR | /bla'tur/ | /bla'tur/ | C |
| PORMETÓ | /porme'to/ | /por'meto/ | E |
| FUCARA | /fu'cara/ | /'fucara/ | E |
| CARANÓN | /kara'non/ | /ka'ranon/ | E |

C, E: correct, error.

**Deleted accent words reading**

| Source words | | Deleted accent words | | MM answers | |
|---|---|---|---|---|---|
| Words | Pronunciation | Stimulus | Pronunciation | Answers | Score |
| OÍDO | /o’ido/ | OIDO | /’oido/ | /o’ido/ | E |
| UNIÓN | /uni’on/ | UNION | /’union/ | /uni’on/ | E |
| LÓGICA | /’loxica/ | LOGICA | /loxica/ | /’loxica/ | E |
| MÚSICA | /’musica/ | MUSICA | /mu’sica/ | /’musica/ | E |
| DIRECCIÓN | /direk’sion/ | DIRECCION | /di’reksion/ | /direk’sion/ | E |
| MANÍ | /ma’ni/ | MANI | /’mani/ | /’mani/ | C |
| PÚGIL | /’pugil/ | PUGIL | /pu’gil/ | /pu’gil/ | C |
| BÓLIDO | /’bolido/ | BOLIDO | /bo’lido/ | /bo’lido/ | C |
| OBÚS | /o’bus/ | OBUS | /’obus/ | /’obus/ | C |

C, E: correct, error.
All errors were stress “lexicalization”.
